# Effects of Post Annealing Treatments on the Interfacial Chemical Properties and Band Alignment of AlN/Si Structure Prepared by Atomic Layer Deposition

**DOI:** 10.1186/s11671-016-1822-x

**Published:** 2017-02-08

**Authors:** Long Sun, Hong-Liang Lu, Hong-Yan Chen, Tao Wang, Xin-Ming Ji, Wen-Jun Liu, Dongxu Zhao, Anjana Devi, Shi-Jin Ding, David Wei Zhang

**Affiliations:** 1 State Key Laboratory of ASIC and System, Institute of Advanced Nanodevices, School of Microelectronics, Fudan University, Shanghai, 200433 China; 2 State Key Laboratory of Luminescence and Applications, Changchun Institute of Optics, Fine Mechanics and Physics, Chinese Academy of Sciences, Changchun, 130033 China; 3 Inorganic Materials Chemistry, Ruhr-University Bochum, 44780 Bochum, Germany

## Abstract

The influences of annealing temperature in N_2_ atmosphere on interfacial chemical properties and band alignment of AlN/Si structure deposited by atomic layer deposition have been investigated based on x-ray photoelectron spectroscopy and spectroscopic ellipsometry. It is found that more oxygen incorporated into AlN film with the increasing annealing temperature, resulting from a little residual H_2_O in N_2_ atmosphere reacting with AlN film during the annealing treatment. Accordingly, the Si–N bonding at the interface gradually transforms to Si–O bonding with the increasing temperature due to the diffusion of oxygen from AlN film to the Si substrate. Specially, the Si–O–Al bonding state can be detected in the 900 °C-annealed sample. Furthermore, it is determined that the band gap and valence band offset increase with increasing annealing temperature.

## Background

AlN is considered to be a promising semiconductor material because of its wide direct band gap, high piezoelectric response and small mismatches of lattice constant, and thermal expansion coefficient with Si substrate. It is these advantages of AlN material that has made it an attractive candidate material for numerous applications in various optoelectronic and microelectronic devices on Si substrate [1–3]. Bulk AlN has a high dielectric constant of 9, and the interface between AlN and Si is established to be thermally stable. Preparing AlN/Si metal-insulator-semiconductor (MIS) structures with acceptable qualities will thus open the path to co-integration of light emitting nitrides with silicon microelectronics [4, 5]. Silicon field-effect transistors using AlN as gate dielectric have been demonstrated by several studies with good performance [6, 7]. So far, many deposition methods have been pursued to obtain AlN films, such as molecular beam expitaxy (MBE) [8], metal-organic chemical vapor deposition (MOCVD) [9], and sputtering [10]. Considering a promising deposition method for AlN film, atomic layer deposition (ALD) has its unique advantage in uniformity, conformality, and thermal budget. Besides these properties mentioned above, ALD is also an excellent deposition method to control the thickness of the AlN film at a single atom layer scale.

In addition, AlON is also a promising material as gate insulator with a dielectric constant as high as 13. Furthermore, it is reported that the AlON layer can provide a prevention of silicon diffusion to the gate insulator [11, 12]. Inserting AlON layer between HfO_2_ gate oxide and Si substrate can suppress the interlayer between AlON and Si resulting in a high quality channel layer [13, 14]. As is known, film properties of AlON can be tailored between those of pure aluminum nitride (AlN) and aluminum oxide (Al_2_O_3_), depending on different applications [15].

Experimentally, the as-deposited AlN thin films usually not only possess a little of oxygen, but also easily form AlON compounds with the subsequent high temperature thermal treatments [16–18]. However, the annealing temperature dependence on the interfacial chemical properties and band alignment of the ALD-AlN/Si structure has not been thoroughly investigated, which plays an important part in the electronic properties of the corresponding device. As a result, thermal ALD system is used to grow thin AlN film on Si substrate in this work. The effect of annealing temperature under N_2_ ambient on the interfacial chemical bonding states of the obtained AlN/Si structure have been investigated by x-ray photoelectron spectroscopy (XPS) in detail. Furthermore, the electronic energy band alignment of the heterojunction structure is also determined as a function of N_2_ annealing temperature.

## Methods

Thin AlN film with 70 cycles (~6.5 nm) was deposited on p-type silicon wafer with crystal orientation of (100) and sheet resistivity of 2~4 Ω cm using atomic layer deposition (ALD) at 370 °C. The precursors trimethylaluminum (TMA) and NH_3_ have been utilized as Al and N sources, respectively. Prior to the growth of AlN films, the Si substrates were cleaned using the Radio Corporation of America (RCA) cleaning process and then dipped into a 2% hydrofluoric acid (HF) aqueous solution for 2 min to remove the native oxides. To investigate the effect of the high temperature annealing on the interfacial chemical properties of the AlN/Si structure, ex situ post-deposition rapid thermal annealing (RTA) was conducted under N_2_ ambient condition with a temperature range of 600–900 °C for 60 s. An ex-situ SPECS XPS system with a monochromatic Al *Kα* source (*hv =* 1486.6 eV) for the excitation of photoelectrons was utilized for data acquisition. The source power was 150 W (10 kV, 15 mA) and the analysis region was a round spot with a radius of 500 *μ*m, an incident angle of 58^o^, and a takeoff angle of 90^o^. Broad band scans with a step of 0.5 eV and pass energy of 25 eV are performed twice in order to acquire the binding energy of specific elements. Charge neutralization was performed with an electron flood gun. Narrow scans with a step of 0.05 eV and pass energy of 25 eV are performed for 20 times for binding energy of specific elements. Furthermore, the valence band scans with a step of 0.05 eV and pass energy of 25 eV are performed for 30 times to obtain the valence band spectra. Charge correction is performed using the position of C 1 *s* spectra at 284.6 eV. Moreover, the XPS spectrometer energy scale was calibrated using Ag 3*d*_5/2_, Cu 2*p*_3/2_, and Au 4*f*_7/2_ photoelectron lines located at 368.06, 932.47, and 83.78 eV, respectively. Spectral deconvolution was performed by Shirley background subtraction using a Voigt function convoluting the Gaussian and Lorentzian functions. Spectroscopy ellipsometry measurements were also performed using a commercial instrument (Sopra GES5E, SOPRA, Courbevoie, France) to obtain the band gap and thickness of AlN film where the incident angle was fixed at 75°, and the wavelength region from 190 to 900 nm was scanned with 5-nm steps.

## Results and Discussion

**Fig. 1:**
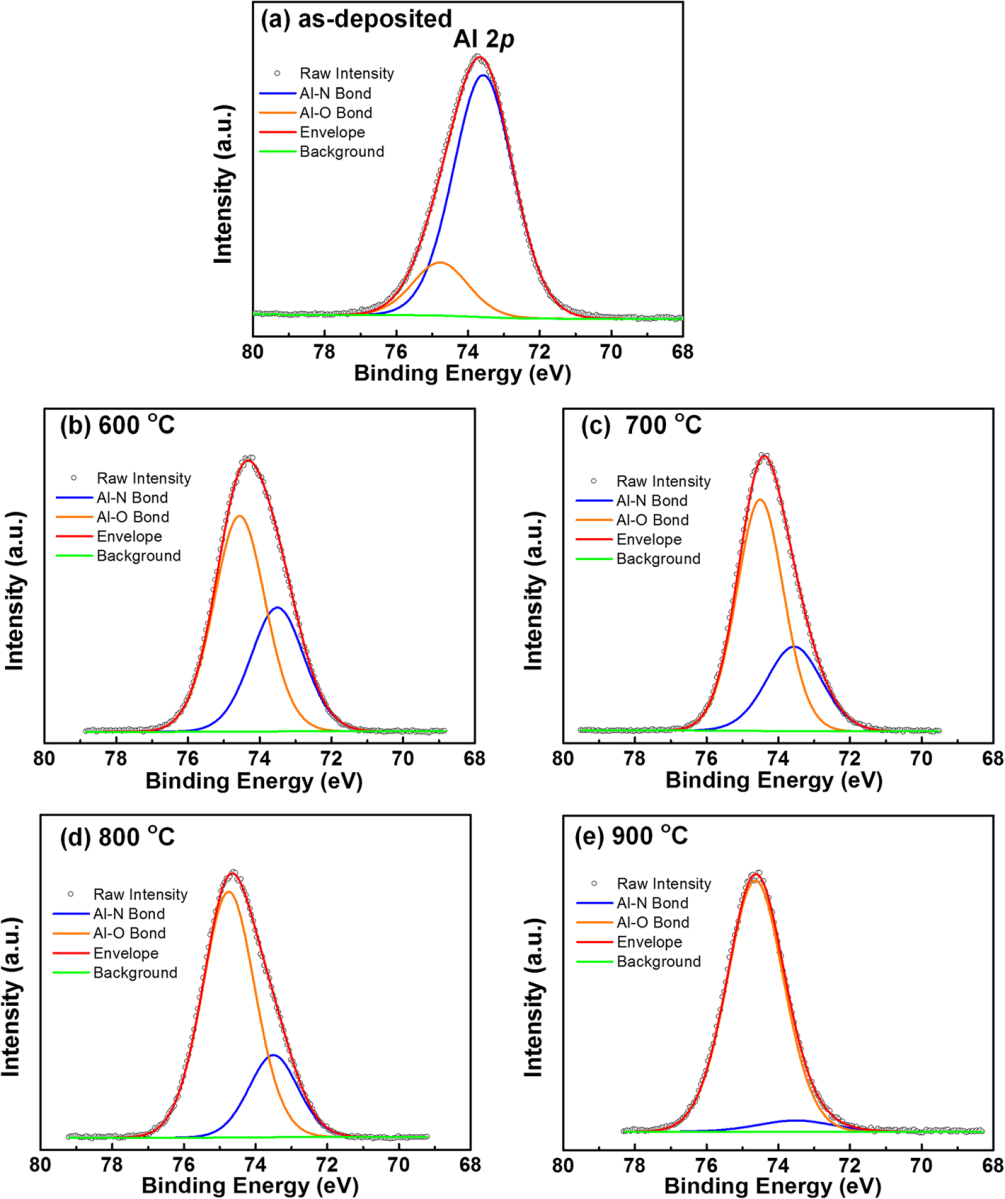
Al 2*p* photoelectron spectra of AlN/Si structures: **a** as-deposited, **b** annealed at 600 °C, **c** annealed at 700 °C, **d** annealed at 800 °C, and **e** annealed at 900 °C for 60 s in N_2_ ambient

**Fig. 2:**
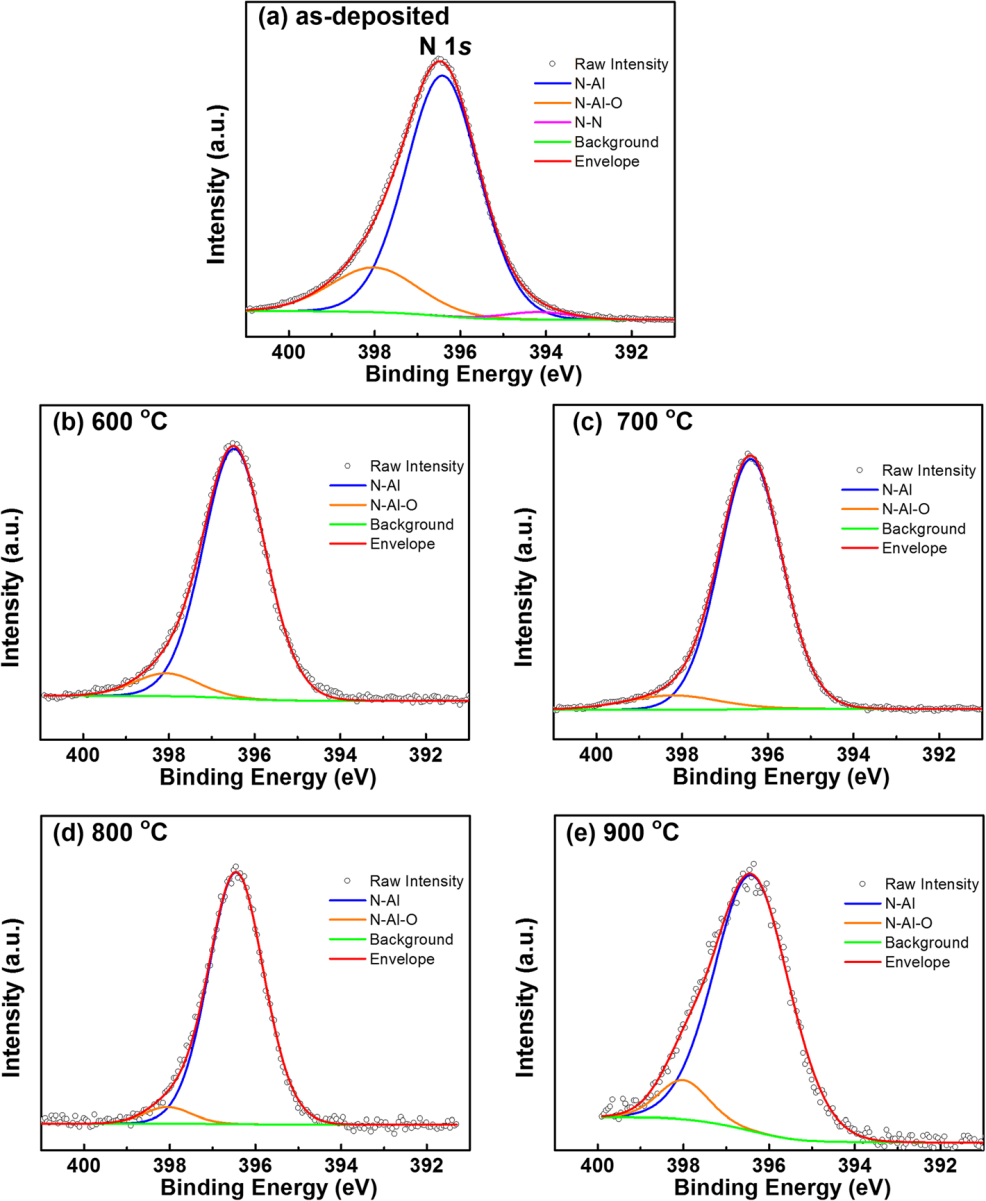
N 1 *s* photoelectron spectra of AlN/Si structures: **a** as-deposited, **b** annealed at 600 °C, **c** annealed at 700 °C, **d** annealed at 800 °C, and **e** annealed at 900 °C for 60 s in N_2_ ambient

In order to investigate the evolution of the interfacial chemical states of AlN/Si structures related with the annealing temperature, the profiles of Al, N, and Si chemical states were examined using XPS. Figure 1 shows the Al 2*p* XPS spectra with the thermal annealing treatment. It is evident that the Al 2*p* for all of the samples can be fitted by two subpeaks located at 73.5 ± 0.1 and 74.6 ± 0.1 eV, corresponding to Al–N and Al–O bonds, respectively. The binding energy positions are in good agreement with the reported results [19]. For the as-deposited sample, the Al–O bonds can be attributed to surface oxidation of AlN layer due to the exposure to air during transporting the sample to the XPS system. Similar results were reported for AlN films deposited by PEALD using NH_3_ plasma on Si substrate [20]. Moreover, the intensity of the Al–N subpeak attenuates and the Al–O subpeak increases relatively with increasing the annealing temperature, suggesting that more oxygen incorporated into AlN film, leading to formation of more Al–O at higher annealing temperature in the N_2_ ambient. The oxygen concentration is originated from the trace amounts of residual H_2_O contained in the N_2_ atmosphere. The increase in the Al–O/Al–N intensity ratio after RTA can be attributed to the breakage of Al–N bonds which are metastable with respect to O. In order to understand the oxidation of AlN film, the atomic ratio of N/Al was calculated to be 0.76, 0.32, 0.31, 0.23, and 0.14 for the as-deposited, 600, 700, 800, and 900 °C annealed sample, respectively. It suggests that the breakage of Al–N bonds is more intense with the increasing annealing temperature. This is in good agreement with reported results, where a strong N loss from the previously N-rich AlN film was observed after vacuum and O_2_ RTAs [11]. Figure 2 shows the N 1 *s* spectra of the as-deposited and subsequently annealed AlN samples at 600, 700, 800, and 900 °C, respectively. For the as-deposited sample, the fitted three peaks located at binding energies of 396.4, 398.0, and 394.6 eV are attributed to N–Al, N–Al–O, and N–N bonding states, respectively. It shall be noticed that the dominant N–Al bonding and N–Al–O bonding can be detected in all the samples, but the N–N bonding only exists in the as-deposited one. With the annealing temperature increasing, the N–Al–O subpeak is observed to decrease in intensity as the annealing temperature increases. It suggests that the three-component system of N–Al–O decomposed into Al–N and Al–O under higher temperature. This is in good accordance with the result of the situation of AlN/AlGaN/GaN system annealing in N_2_ atmosphere [21]. In fact, both N–Al and N–Al–O subpeaks are observed to decrease in intensity as the annealing temperature increases. Nevertheless, the decomposition of N–Al bonds is more intense than the one of N–Al–O when the annealing temperature reaches to 900 °C. Therefore, it looks like the intensity of N–Al–O bonds enhanced relative to the N–Al bonds.

**Fig. 3:**
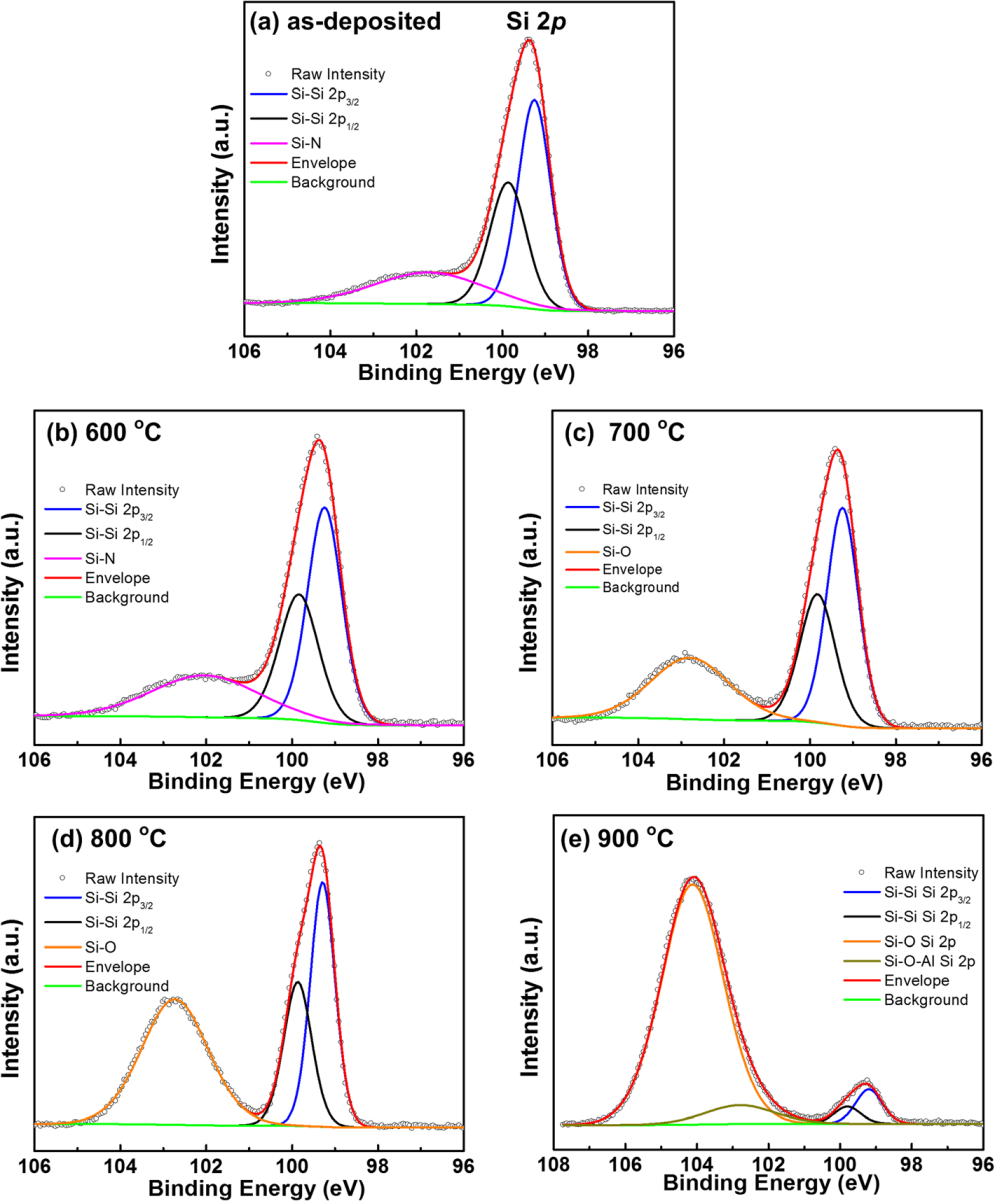
Si 2*p* photoelectron spectra of AlN/Si structures. **a** As-deposited, **b** annealed at 600 °C, **c** annealed at 700 °C, **d** annealed at 800 °C, and **e** annealed at 900 °C for 60 s in N_2_ ambient

The Si 2*p* core-level spectra for the AlN/Si structures taken at normal emission setup for as-deposited and annealing samples are shown in Fig. 3. It can be noted for all the samples that the subpeak located at lower binding energy side is composed of two spin-orbit split peaks Si 2*p*_1/2_ and Si 2*p*_3/2_ owing to Si substrate. The two peaks are located at 99.8 and 99.2 eV, respectively, with a spin orbit split (S.O.S) of 0.6 eV and an intensity ratio of 1:2. For the as-deposited sample and 600 °C annealing sample, the Si 2*p* subpeak located at 101.8 eV is correlated to Si–N bond states, which is in good agreement with the reported results [22]. The Si–N bonding at the interface of AlN/Si heterostructure is beneficial to the surface passivation of the Si substrate and proved to be chemically stable. When the annealing temperature increases to 700 and 800 °C, it can be seen that the Si–N bonding disappears completely. However, a new Si 2*p* subpeak with a binding energy of 102.7 ± 0.1 eV can be detected, which can be assigned to the formed interfacial SiO_x_ (*x* < 2) layer during thermal annealing [23]. With the increase of annealing temperature from 700 to 800 °C, the intensity of Si–O subpeak increases and shifts toward high binding energy sides, resulting from the complete oxidation of the suboxides involved in the interfacial layer. The formation of Si–O bonds during annealing treatment is caused by the diffusion of the oxygen contained in the AlN film and the trace amount of residual H_2_O contained in the N_2_ atmosphere. Comparing the 600 °C annealing sample with the as-deposited sample, it can be seen that the Al–O/Al–N intensity ratio increases dramatically. But for Si 2*p* spectrum, there are still Si–N bondings but not any Si–O bonds at the interface, suggesting that at 600 °C the AlN film protected the Si substrate from oxidation near the interface. In addition, after the sample annealed at 900 °C, another new peak at 103.33 eV corresponding to the Si–O–Al bonds can be found, confirming the formation of silicate layer. It suggests that the interfacial property of AlN/Si structure has degraded significantly after 900 °C annealing [24].

**Fig. 4:**
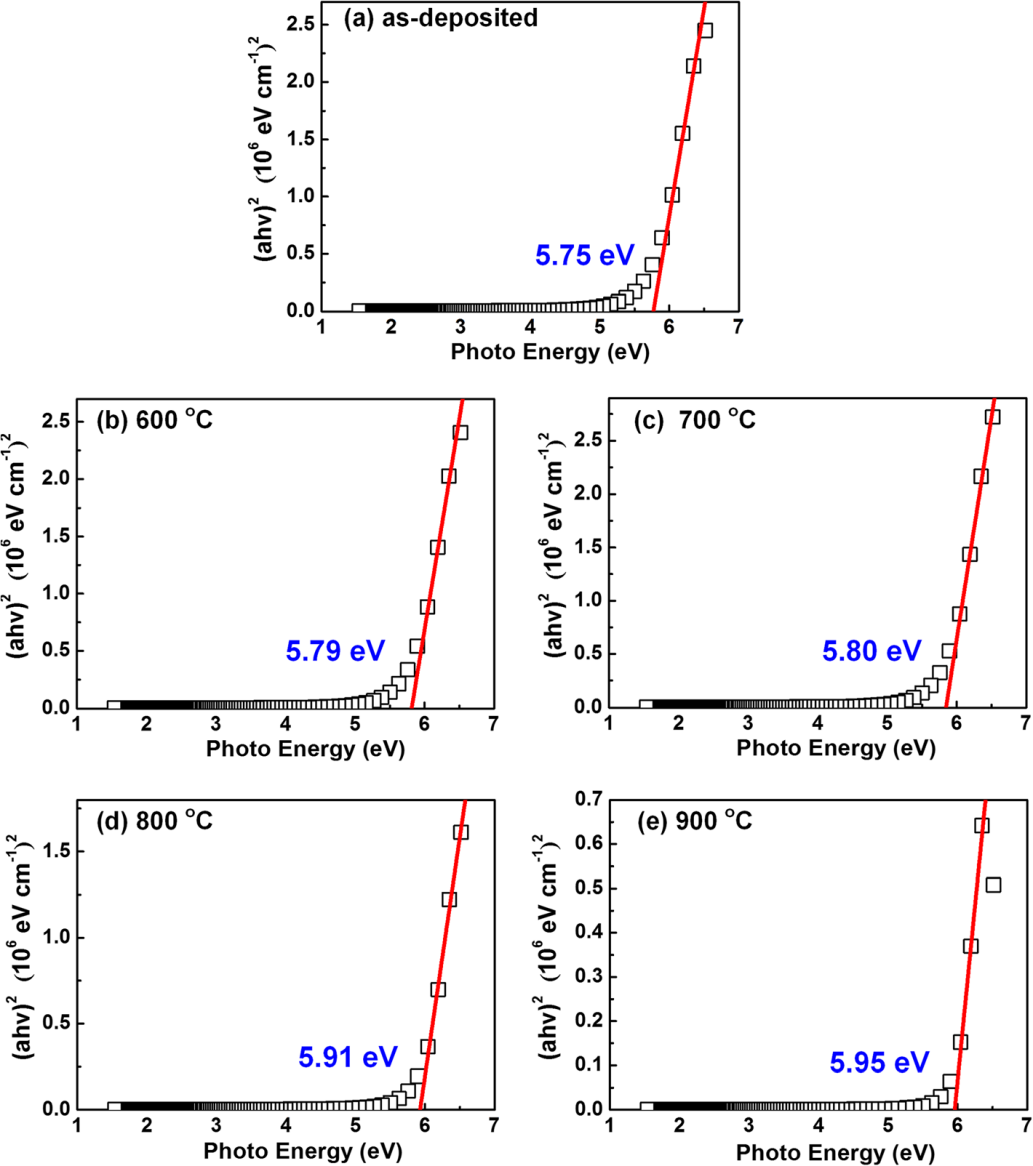
The bandgap energy of AlN films under various annealing temperature

In addition to the interfacial analysis, the band offsets of the AlN/Si samples with thermal treatment are also investigated based on the fact that the band alignment is one of the most fundamental physical properties for the application of semiconductor device. To determine the band offsets of the AlN/Si under different annealing temperatures, the bandgap (*E*_g_) of the as-prepared films will be determined firstly. In the case of AlN, the value of *E*_g_ can be extracted from the experimental absorption coefficient assuming a direct transition of electrons from the valence band to the conduction band. Generally, the *E*_g_ can be estimated by using the so-called Tauc’s method [25]

1 $$ {\left(\alpha h\upsilon \right)}^2\propto A\left(E-{E}_{\fontfamily{Calibri Light}{g}}\right) $$

where *α* is the absorption coefficient, *hυ* is the incident photon energy, and *A* is a constant. The absorption coefficient *α* is related to the extinction coefficient (*k*) of the sample via *α* = 4*πk/λ* where *λ* is the wavelength of the incident light. The bandgap energy can be found by drawing relatively a linear fit line with a maximum negative slope in the curve. The tangent intersects with the horizontal axis and the crossing point gives the band gap value. It can be observed from Fig. 4, the band energies of the as-deposited and annealed samples from 600 to 900 °C are 5.75, 5.79, 5.80, 5.9,1 and 5.95 eV, respectively. The bandgap value of Al_2_O_3_ (6.8 eV) is larger than the one of AlN (6.0 eV) [26, 27]. With the increase in annealing temperature, the band energies of AlN increase owning to the incorporation of O element into the AlN film and the formation of Al_2_O_3_. It must be pointed out that the band gap value of as-deposited sample is less than the bulk AlN band gap value. It can be attributed to a variety of crystal defects in the prepared AlN layer, which may induce intermediate energy levels that merge with the conduction band, and lower the bandgap [17].

**Fig. 5:**
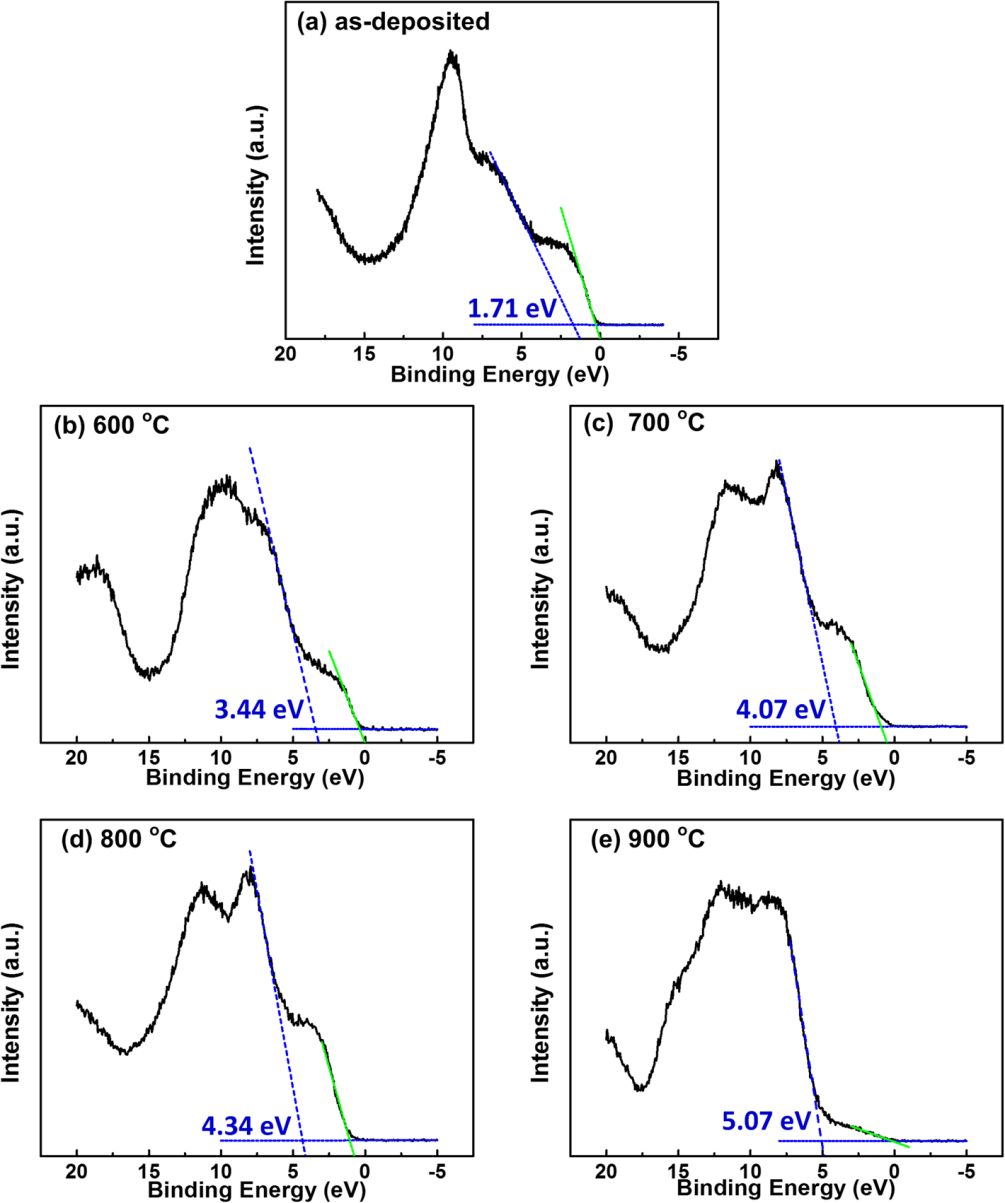
Valence band spectra of AlN/Si structures determined by extrapolation of the leading edge to the base line

**Fig. 6:**
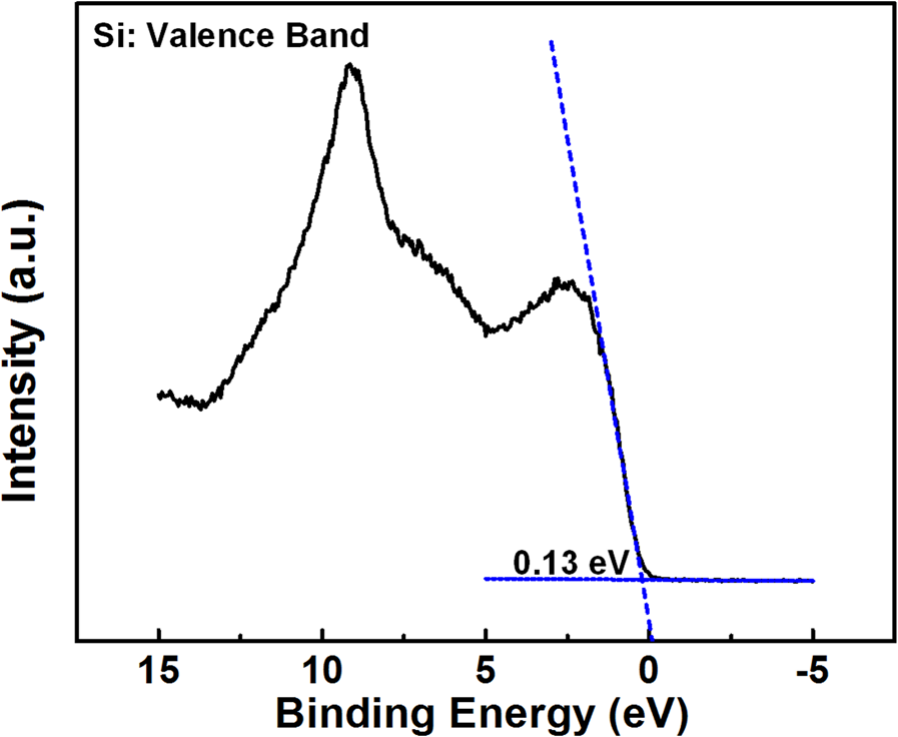
Valence band spectra of bulk Si determined by linear extrapolation method

**Fig. 7:**
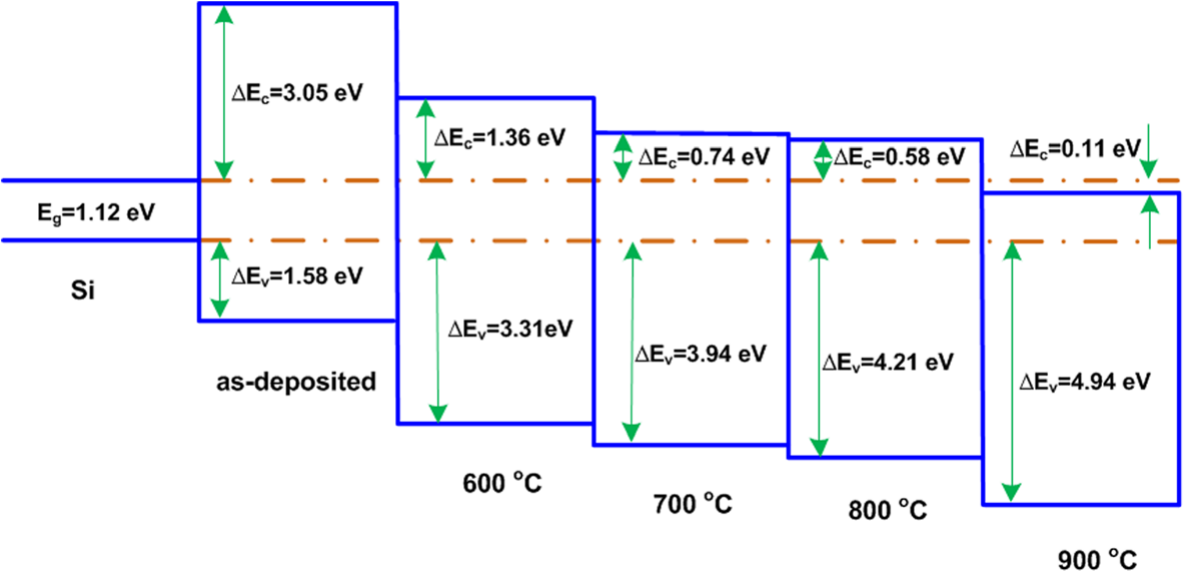
Schematic band diagram of the as-deposited and annealed AlN/Si structures

Figure 5 presents the valence band spectra of AlN/Si structures determined by linear extrapolation method. Two slopes have been observed clearly, in which the slope in the lower binding energy regime is corresponding to the Si valence band onset and the one in the higher energy regime is corresponding to the AlN film valence band onset. The values of the valence band maximum (VBM) for the AlN film deposited on Si substrate under various annealing temperature are then determined to be 1.71, 3.44, 4.07, 4.34, and 5.07 eV, respectively. On the other hand, the valence band spectrum for the Si substrate is found to be located at 0.13 eV in Fig. 6. According to Kraut’s method [28], the valence band offset (ΔE_v_) and the conduction band offset (ΔE_c_) of AlN/Si interface can be described to the following equations:

2 $$ \varDelta\ {E}_v = {E}_{\mathrm{VBM}}^{\mathrm{film}}-{E}_{\mathrm{VBM}}^{\mathrm{Si}} $$

3 $$ \varDelta\ {E}_c = {E}_{\fontfamily{Calibri Light}{g}}^{\mathrm{film}}-{E}_{\fontfamily{Calibri Light}{g}}^{\mathrm{Si}}-{E}_v^{\mathrm{film}/\mathrm{S}\mathrm{i}} $$

where ${E}_{\mathrm{VBM}}^{\mathrm{film}}$ is the valence band maximum of AlN, ${E}_{\mathrm{VBM}}^{\mathrm{Si}}$ is the valence band maximum of bulk Si, ${E}_{\fontfamily{Calibri Light}{g}}^{\mathrm{film}}$ is the band gap of AlN, ${E}_v^{\mathrm{film}/\mathrm{S}\mathrm{i}}$ is the valence band offset, and ${E}_{\fontfamily{Calibri Light}{g}}^{\mathrm{Si}}$ band gap of the Si wafer. Based on the above equations, the valence band offset for the as-deposited AlN film is determined to be 1.58 eV. With the increase of the annealing temperature, the valence band offsets of 3.31, 3.94, 4.21, and 4.94 eV for the annealed samples on Si substrate has been also detected, respectively. It is observed that the valence band offset shifts toward higher energy with increasing the annealing temperature. As is well known, the predominant contribution to the AlN valence band is from the N 2*p* orbital [29], and the valence band maximum of Al_2_O_3_ is mainly determined by O 2*p* [30]. Therefore, the shift toward higher energy in the valence band maximum of AlN film with the annealing temperature is mainly on account of the generation of Al_2_O_3_. With the knowledge of the Si band gap value of 1.12 eV, ΔE_c_ values for as-deposited and annealed from 600 to 900 °C samples are calculated to be 3.05, 1.36, 0.74, 0.58, and −0.11 eV, respectively. Figure 7 shows a summary of the schematic band diagram of the as-deposited and annealed AlN/Si structures at different temperatures. For the as-deposited and 600 °C annealed sample, both ∆E_c_ and ∆E_v_ values are over 1 eV, which satisfies the requirement to guarantee the sufficient barrier height in metal-insulator-semiconductor (MIS) application. When the temperature comes to 700 and 800 °C, and the ΔE_c_ values are all found to be less than 1 eV, which could be utilized in optoelectronic device applications. Furthermore, for 900 °C annealed sample, the band structure changed to be type II band alignment. The feasibility to tune the band alignment of AlN/Si structure by selecting a proper annealing temperature shows advantage for AlN/Si structure used in different applications,where requires different band alignment types.

## Conclusions

In summary, the influences of annealing temperature in N_2_ atmosphere on interfacial chemical properties and band alignment of AlN/Si structure prepared by thermal atomic layer deposition have been investigated. It has been found that more oxygen incorporated into AlN film with the increasing annealing temperature, resulting from a little residual H_2_O existed in N_2_ atmosphere reacting with AlN film during the annealing process. The Si–N bonding at the AlN/Si interface gradually transforms to Si–O bonding with the increasing temperature, due to the diffusion of oxygen from AlN film to the Si substrate. The band gap and valence band offset increase with increasing annealing temperature. These results indicate that the modification of the band structure of the AlN/Si heterojunction can be realized by properly selecting the annealing temperature.
